# COMT and 5-HT1A-receptor genotypes potentially affect executive functions improvement after cognitive remediation in schizophrenia

**DOI:** 10.1080/21642850.2014.905206

**Published:** 2014-04-28

**Authors:** Marta Bosia, Margherita Bechi, Adele Pirovano, Mariachiara Buonocore, Cristina Lorenzi, Federica Cocchi, Placido Bramanti, Enrico Smeraldi, Roberto Cavallaro

**Affiliations:** ^a^ Clinical Neurosciences, San Raffaele Scientific Institute, Via Stamira d'Ancona 20, Milan20127Italy; ^b^ Center for Neurolinguistics and Theoretical Syntax, Institute for Advanced Study, Pavia, Italy; ^c^ Faculty of Medicine and Surgery, Università Vita-Salute San Raffaele, Milan, Italy; ^d^ IRCCS Centro Neurolesi “Bonino Pulejo”, Messina, Italy

**Keywords:** genomics, mental health and disorder, rehabilitation, cognitive behaviour therapy, psychometrics

## Abstract

Cognitive remediation therapy (CRT) has been proved to improve cognitive deficits in schizophrenia and to enhance functional outcomes of classical rehabilitation. However, CRT outcomes are heterogeneous and predictors of response are still unknown. Genetic variability, especially in the dopaminergic system, has been hypothesized to affect CRT. We previously reported that rs4680 of the catechol-O-methyltrasferase (COMT) influences improvements in executive functions in patients treated with CRT, but this result was not confirmed by other studies. Such inconsistent findings may depend, other than on clinical variables, also on other genes involved in cognition. Recent studies proved that serotonin 1A receptor (5-HT1A-R) regulates dopamine in the prefrontal cortex (PFC), and clinical works suggested a 5-HT1A-R role in cognition. We then analysed possible effects of COMT rs4680 and 5-HT1A-R rs6295 on CRT outcomes, taking into account also clinical and demographic factors. Eighty-six clinically stabilized schizophrenia patients treated with three months CRT were assessed with the Wisconsin Card Sorting Test, as a measure of executive functions, at enrolment and after CRT treatment, and underwent COMT and 5-HT1A-R genotyping. We found a significant main effect of COMT genotype and an interaction with 5-HT1A-R on executive function improvement after CRT. The results suggest that these two polymorphisms may have an additive effect on individual capacity to recover from cognitive deficit, probably through their role on PFC dopaminergic transmission modulation, known to be critical for modulating cognitive functions.

## 1. Introduction

In recent years, schizophrenia has increasingly been recognized as a neurocognitive disorder, leading to a growing literature on new deficit-specific therapeutic strategies. Among these, cognitive remediation treatment (CRT), an intensive training including exercises created to target impaired cognitive functions, has been proved to improve neurocognitive domains, such as executive functions and to enhance functional outcomes of classical rehabilitation (Bell, Bryson, Greig, Corcoran, & Wexler, 2001; Bellucci, Glaberman, & Haslam, 2003; Cavallaro et al., 2009; Wykes, Reeder, Corner, Williams, & Everitt, 1999). However, the degree of improvement after CRT is still heterogeneous and this variability could rely on many factors, including demographic, symptoms and cognitive variables and also genetic polymorphisms (Wykes, Huddy, Cellard, McGurk, & Czobor, 2011; Wykes & Spaulding, 2011).

The focus is on genes involved in the prefrontal cortex (PFC) dopamine transmission, known to be critical for core cognitive performances impaired in schizophrenia and showing decreased activation efficiency in patients (Manoach, 2003).

A major role in dopamine levels regulation in the PFC is played by catechol-O-methyltrasferase (COMT), an enzyme for dopamine degradation. The COMT gene presents a functional polymorphism (rs4680) consisting of an A to G substitution, at the codon 158, resulting in the presence of valine instead of methionine, leading to a three to four times higher enzymatic activity and therefore lower dopamine levels (Chen et al., 2004). There is large consensus in the literature of an association between COMT Met allele and “better” PFC function, evaluated by means of both related performances through executive functions tests and regional activation through functional imaging studies (Bertolino et al., 2006; Kurnianingsih et al., 2011). Moreover, we previously reported a significant enhancing effect of cognitive remediation and COMT rs4680 polymorphism on executive functions improvement in schizophrenia patients, suggesting a genetic interaction also on dynamic modulation of cognitive performances and thus on patient's ability to recover from deficit (Bosia et al., 2007).

Recent studies outlined the role of serotonin 1A Receptor (5HT1A-R) in dopamine regulation, particularly in the PFC. Systemic administration of 5-HT1A-R agonists has been reported to increase dopamine release in the PFC and this is blocked by treatment with the 5-HT1A antagonist (Rollema, Lu, Schmidt, Sprouse, & Zorn, 2000). However, the direction of agonist effects seems to be dose-dependent, Diaz-Mataix, Artigas, and Celada (2006) observed that reverse dialysis of the 5-HT1A-R agonist BAYx3702 produced a biphasic effect on PFC dopamine in mouse and rat: a low concentration increased, while a higher concentration decreased dopamine. The authors suggested that this effect probably depends upon whether presynaptic 5-HT1A autoreceptors in the raphe are preferentially stimulated or postsynaptic 5-HT1A-R are also engaged. 5-HT1A-R has also been claimed to play a role in cognition. There is extensive literature in rodents suggestive of pro-cognitive properties of 5-HT1A-R ligands in model of working, episodic, reference, and/or spatial memory, and concerning both acquisition and/or retention (Meneses, 1999; Steckler & Sahgal, 1995) and of cognitive flexibility in a reversal learning task (Depoortere et al., 2007). In schizophrenia patients, Sumiyoshi et al. (2001) observed a significant improvement in executive functions and verbal memory after six weeks of treatment with tandospirone, a 5-HT1A-R agonist, adjunctive to neuroleptic. A functional variant in the promoter region of the 5-HT1A-R gene was reported (rs6295); this polymorphism consists of a C to G substitution at position –1019, modulating the rate of transcription of 5HT1A-R gene, the mutated G allele resulting in higher transcriptional levels (Lemonde et al., 2003). An effect of this genetic variant has been observed on a complex cognitive task of Theory of Mind in patients with schizophrenia (Bosia et al., 2011).

Based on this rationale, we hypothesized that 5-HT1A-R rs6295 and COMT rs4680, both involved in regulation of PFC dopaminergic transmission, could affect cognitive remediation outcomes in schizophrenia. We aimed to evaluate possible predictive effects of 5-HT1A-R rs6295 and COMT rs4680, including main effects and cumulative effect, on executive functions’ improvement after CRTs, also taking into account clinical factors.

## 2. Methods

### 2.1. Sample

The study group included 86 biologically unrelated, clinically stabilized outpatients meeting DSM-IV criteria for schizophrenia. All subjects provided informed consent to a protocol approved by the local Ethical Committee and following the principles of the Declaration of Helsinki.

### 2.2. Genotyping

Genotypic analysis for COMT Val158Met and 5-HT1A-R – 1019C/G polymorphisms was performed as previously described in other studies by our group (Bosia et al., 2007; Lorenzi et al., 2005).

In detail, for COMT (rs4680), DNA was extracted from whole blood by manual extraction, using the “Illustra blood genomicPrep Midi Flow kit” (GE Healthcare, Milan, Italy). Polymerase chain reaction (PCR) was performed with the following primers: 5 ACT GTG GCT ACT CAG CTG TG 3, 5 CCT TTT TCC AGG TCT GACAA 3. The PCR reaction was carried out by using the ABI 9700 PCR thermal-cycler (Applied Biosystems, APPLERA) in a 10 µl volume containing 150 ng of genomic DNA, 5 pmol of each primer, 10 nmol of dNTPs mix with 7-deaza-dGTP, 10× HotMaster Taq Buffer, 0.5 µl of DMSO and 1 U of HotMaster Taq DNA Polymerase (Eppendorf, Milan, Italy). The PCR reaction was performed by using the ABI 9700 PCR thermal-cycler (Applied Biosystems, APPLERA) as follows: after a first step at 94°C for 6 min, steps of 94°C for 75 s, 58°C for 25 s and 70°C for 70 s for 35 cycles. Then, a final extension step at 70°C for 10 min was added. The amplified fragment was then purified by using the Multi-Screen Colum Loader (MILLIPORE), filled up and packaged with Sephadex G-50 (Sigma-Aldrich's) to remove residual PCR reagents. An aliquot of the purified PCR product was then used to perform sequencing reaction, using the DYEnamic ET Dye Terminator Cycle Sequencing Kit (GE Healthcare, Milan, Italy). After a new purification step, the fragment was then genotyped by using the MegaBACE 500 genetic analyzer (GE Healthcare, Milan, Italy) under standard conditions.

For 5-HT1A-R (rs6295), DNA was extracted from whole blood by manual extraction, using the “Illustra blood genomicPrep Midi Flow kit” (GE Healthcare, Milan, Italy). The rs6295 polymorphism is located in the promoter region of the 5-HTR1A gene and consists of a C to G substitution at position 92928 (GDB: AC008965). It is inside a palindromic region of 26 bp, which bounds a single repressor, the so-called nuclear DEAF-1-related protein. PCR was performed with the following primers: 5-CCC AGA GTG GCA ATA GGA GA-3 and 5-CCG TTT TGT TGT TGT TGT CG-3. The PCR reaction was carried out in a 10 µl volume containing 150 ng of genomic DNA, 5 pmol of each primer, 10 nmol of dNTPs mix, 10× HotMaster Taq Buffer and 0.5 U of HotMaster Taq DNA Polymerase (Eppendorf, Milan, Italy). The PCR reaction was performed using the ABI 9700 PCR thermal-cycler (Applied Biosystems, APPLERA) as follows: after a first step at 94°C for 5 min, steps of 94°C for 35 s, 62°C for 35 s and 70°C for 45 s for 35 cycles. Then, a final extension step at 70°C for 10 min was added. The amplified fragment was then purified using the Multi-Screen Colum Loader (MILLIPORE), filled up and packaged with Sephadex G-50 (Sigma-Aldrich's) to remove residual PCR reagents. An aliquot of the purified PCR product was then used to perform the sequencing reaction, using the DYEnamic ET Dye Terminator Cycle Sequencing Kit (GE Healthcare, Milan, Italy). After a new purification step, the product was then sequenced using the MegaBace 500 genetic analyzer (GE Healthcare, Milan, Italy) under standard conditions.

### 2.3. CRT protocol and assessments

Patients were assigned to a standard rehabilitation treatment added to three months CRT, consisting of three hours a week of domain-specific computerized neurocognitive exercises. The computer-aided training employs the Cogpack Software (Marker, 1987–2007), which includes different neurocognitive exercises that can be divided into domain-specific exercises, aimed at training specific cognitive areas among the ones known to be impaired in schizophrenia and non-domain-specific exercises that do not focus on one specific function. The CRT consists of three one-hour sessions a week of domain-specific computer-aided exercises, for a period of 12 weeks, giving a total of 36 hours. Sets of exercises are individually created for each patient based on the baseline assessment and graded in difficulty, with easier ones being presented earlier in the programme. Exercises are administered by trained psychologists.

At enrolment basic demographic and clinical data were collected and patients were assessed with: Positive and Negative Syndrome Scale (PANSS), a standardized measurement for typological and dimensional symptoms assessment (Kay et al., 1987). It includes 30 items that provide balanced representation of positive and negative symptoms and gauges their relationship to one another and to global psychopathology. The PANSS was administered by trained psychiatrists.

The Wisconsin Card Sorting Test (WCST) (Heaton, 1981) evaluates visuospatial skills and the ability to classify, keep the set, switch the attentive focus and inhibit interfering answers. The test consists in the pairing of coloured cards series, differing in colour, shape and number according to a criterion that changes after a number of consecutive pairings. The performance on the WCST in this study was based on the number of perseverative errors (when the subject keeps pairing the cards according to the previous criterion), previously reported to improve with CRT and to be associated with COMT genotype (Bosia et al., 2007). Perseveration is the measure most clearly associated with dorsolateral PFC defects and is therefore the most frequently assessed in schizophrenia trials. A computerized version of WCST was administered by trained psychologists and repeated after CRT.

The Wechsler Adult Intelligence Scale – R (WAIS-R) (Wechsler, 2006) is a standardized test designed to measure intelligence quotient (IQ) in adults and older adolescents, consisting of six verbal (Information, Digit Span, Vocabulary, Arithmetics, Comprehension and Similarities) and five performance (Picture Completion, Picture Arrangement, Block Design, Digit Symbol and Object Assembly) subtests. The WAIS was administered by trained psychologists.

### 2.4. Data analysis

The STATISTICA Software for Windows, version 8 (StatSoft Inc., Tulsa, OK, USA) was used to perform the statistical analyses.

As in a previous work (Bosia et al., 2007) we grouped patients as Val/Val and Met carriers for COMT genotype and C carriers vs. G/G homozygous for 5HT1A genotype, to better evaluate the effect of the mutated G allele in a relatively small sample.

Differences between COMT and 5-HT1A-R genotypes and demographic, clinical and neuropsychological characteristics were analysed by means of analysis of variance (ANOVA) for quantitative measures (i.e. PANSS scores) or chi-squared test for dichotomic variables (gender, COMT by 5HT1A genotype).

Differences between COMT and 5-HT1A-R genotypes and WCST performances (number of perseverative errors) at baseline were analysed by means of analysis of covariance (ANCOVA), with age and number of years of education as covariates, since these variables were found to influence WCST performances in schizophrenia (Banno et al., 2012).

Associations between demographic/clinical characteristics and WCST performances were examined using standard tests of correlation (Pearson *r)*.

To evaluate changes in executive functions after CRT, we calculated an index value, determined by change in the WCST, number of perseverative errors score, divided by the standard error of the whole sample (mean between baseline and post-CRT). This value represents a proxy effect size measure of improvement (Wykes et al., 1999).

A general linear model analysis, with COMT and 5-HT1A-R genotypes as categorical predictors; age, years of education, duration of illness, PANSS negative score, Total IQ and WCST basal performances as continuous predictors and the effect size of improvement on WCST as the dependent variable was used to evaluate possible predictive effects on cognitive improvement after CRT. Fisher *post hoc* test was used to determine significant differences between genotypes groups.

## 3. Results

DNA analysis showed an allelic distribution according to the Hardy–Weinberg equilibrium for both genotypes considered: 15 patients C/C, 50 C/G and 21 G/G for the 5HT1A genotype and 25 G/G, 39 G/A and 22 AA for the COMT genotype. The chi-squared test showed no significant differences in COMT genotype distribution among 5HT1A genotype groups.

All patients were on antipsychotic monotherapy at a stable dose, since at least three months: 50 patients were taking clozapine, 22 risperidone, 10 haloperidol, 3 olanzapine and 1 aripiprazole.

Demographic and clinical characteristics stratified by COMT and 5-HT1A-R genotypes are reported in Table 1. No significant differences were observed between genotype groups.

**Table 1.** Demographic, clinical and neuropsychological features of the sample, stratified by COMT and 5-HT1A-R genotypes.

COMT rs4680	Val/Val	Val/Val	Met carriers	Met carriers	ANOVA/
5-HT1A-R rs6295	G/G	C carriers	G/G	C carriers	Chi-squared
	(Mean ± SD)	(Mean ± SD)	(Mean ± SD)	(Mean ± SD)	
Gender	M* *= 4; F* *= 4	M* *= 7; F* *= 6	M* *= 12; F* *= 5	M* *= 30; F* *= 18	N.S.
Age (yrs)	31.67 ± 4.05	36.53 ± 2.56	34.29 ± 2.41	33.62 ± 1.40	N.S.
Education (yrs)	12.5 ± 0.93	11.54 ± 2.18	10.76 ± 2.93	11.98 ± 3.04	N.S.
Onset (yrs)	22.12 ± 4.45	27.85 ± 11.46	24.53 ± 6.46	23.13 ± 5.36	N.S.
Duration of illness (yrs)	6.63 ± 5.10	11.15 ± 7.84	12.41 ± 7.33	9.73 ± 8.25	N.S.
PANSS negative (score)	20.49 ± 2.23	19.55 ± 4.53	19.29 ± 5.35	19.76 ± 5.40	N.S.
PANSS positive (score)	13.22 ± 0.90	13.48 ± 2.41	12.75 ± 2.49	13.88 ± 3.46	N.S.
PANSS general (score)	33.60 ± 2.99	32.24 ± 4.92	32.22 ± 4.71	32.54 ± 5.01	N.S.
PANSS total (score)	67.29 ± 4.33	65.28 ± 11.13	64.26 ± 9.56	66.18 ± 10.89	N.S.
Total I.Q.	85.63 ± 11.29	86.89 ± 9.58	84.45 ± 12.91	86.44 ± 13.07	N.S.
WCST pers. errors	23.87 ± 12.29	15.00 ± 8.14	15.41 ± 9.91	16.69 ± 11.08	N.S.(Ancova)

Note: yrs, years; SD, standard deviation; N.S., not significant; M, males; F, females.

The ANCOVA did not show any significant effects (main nor interaction) of genotypes on the baseline WCST performances.

Correlation analyses between WCST performances and clinical/demographic variables showed a significant correlation only with PANSS negative score (*r *= .27, *p *< .012).

**Figure 1.**
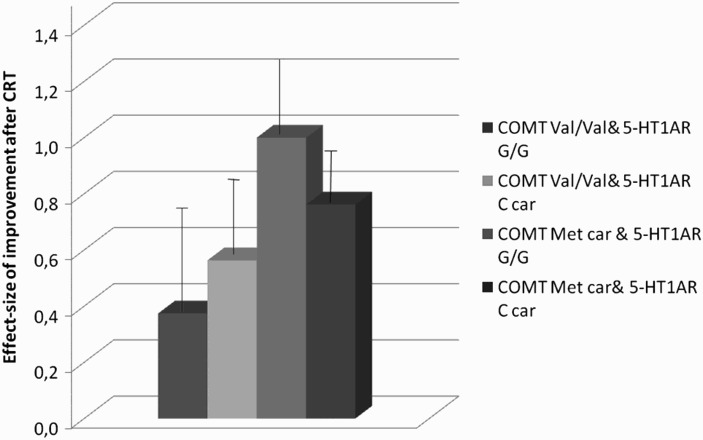
Mean effect size of improvement in executive functions, evaluated by means of WCST number of perseverative errors, stratified by COMT and 5-HT1A-R genotypes.

The general linear model analysis revealed a significant overall model (*R* ^2^ **= .76, *F *= 9.89, *p*< .0001). Significant main effects were observed for years of education (*F *= 5.04, *p *< .033), WCST basal performances (*F *= 55.26, *p *< .0001) and COMT genotype (*F *= 4.42, *p *< .045). A significant interaction between COMT and 5-HT1A-R genotypes was also reported (*F *= 5.49, *p *< .026). Fisher LSD *post hoc* showed a significant difference (*p *< .039) between groups: the COMT Val/Val – 5-HT1A-R G/G group had a lower improvement in executive functions than the COMT Met Carriers – 5-HT1A G/G group. Mean scores between genotype groups are shown in Figure 1.

## 4. Discussion

Unravelling clinical and biological factors associated with cognitive improvement after CRT may be useful both to help design more personalized interventions and to gain a deeper knowledge of the features underlying cognition.

To our knowledge, this is the first study investigating the interaction between COMT rs4680 and 5-HT1A-R rs6295 polymorphisms in predicting the effect of CRT in subjects affected by schizophrenia. In line with previous reports, we observed significant effects of years of education, basal cognitive level and of COMT genotype (Bosia et al., 2007; Wykes et al., 2011) on the cognitive improvement through CRT. Moreover, we found a significant interaction of COMT rs4680 and 5-HT1A-R rs6295 polymorphisms, with patients carrying both COMT Val/Val and 5-HT1A G/G genotype showing a lower improvement on executive performances, after three months of CRT. Even though significant effects were also observed for education level and basal performances, no significant differences for these variables were observed between genotype groups, suggesting a specific role of the genetic polymorphisms. This finding, although preliminary, suggests a possible additive effect of these two functional polymorphisms, both involved in dopamine regulation, on dynamic modulation of executive functions in schizophrenia. It supports extensive literature reporting worse cognitive performances in COMT Val/Val subjects, which are known to have lower PFC dopamine level, while it leads to some speculations about the role of 5-HT1A polymorphism. Activation of the 5-HT1A receptor plays a direct effect on dopamine release (Rollema et al., 2000) in a biphasic manner: low stimulation of 5-HT1A receptor increases, while higher stimulation decreases dopamine in the PFC, due to inhibitory actions on pyramidal glutamatergic cells (Diaz-Mataix et al., 2005). The G/G genotype is associated with an overexpression of the 5-HT1A receptor and thus an increased receptorial stimulation can be hypothesized as a consequence, leading to a reduction in PFC dopamine. This hypothesis is supported by observation of worse performances in subjects with 5-HT1A-R G/G and COMT Val/Val genotype, already reducing dopamine availability. Moreover, the G allele of 5-HT1A-R was previously reported to be associated with poorer performances of Theory of Mind, a complex function requiring integrity of executive domains (Bosia et al., 2011). Indeed, COMT rs4680 has been reported to affect 5-HT1A receptor-binding potential, with Val/Val homozygous showing higher cortical 5-HT1A binding (Baldinger et al., 2013). The critical role of dopamine's availability for successful CRT is also supported by the report of an association between cognitive training effect and change in cortical dopamine D1 receptor binding, with the degree of cognitive improvement being associated with the decrease in D1 binding (McNab et al., 2009). These data suggest that CRT directly affects dopamine transmission and thus in turn CRT outcomes may be critically influenced by genetic variants regulating PFC dopamine levels.

Even if interpretation of these results is only speculative, it suggests again that prefrontal dopamine availability is critical for the patient's ability to recover from cognitive deficit through specific rehabilitation programmes and its final availability may be influenced by the interaction of multiple candidate gene effects. In this view evaluation of specific genetic polymorphisms could become a useful instrument to personalize interventions maximizing the likelihood of successful improvement. Moreover, results suggest that the role of 5-HT1A-R in cognition may be worth further investigation.

However, this study presents some limits that need to be addressed. First, we have a small sample size that prevented us from analysing the three genotype groups separately. Second, we did not include in the analysis a control group treated with placebo instead of CRT, therefore the changes in cognition cannot directly be attributed to CRT. Moreover, we were not able to rule out the possible effect of antipsychotics, which may differently interact with the studied polymorphisms both on dopamine and serotonin transmission. Moreover, we cannot exclude that our result may depend also on other factors, such as different polymorphisms within genes in the same pathways.
